# The Effects of Low-Load Resistance Training Combined with Blood Flow Restriction or Hypoxia on Cardiovascular Response: A Randomized Controlled Trial

**DOI:** 10.3390/life15081162

**Published:** 2025-07-23

**Authors:** Apiwan Manimmanakorn, Pudis Manimmanakorn, Lertwanlop Srisaphonphusitti, Wirakan Sumethanurakkhakun, Peeraporn Nithisup, Nattha Muangritdech, Worrawut Thuwakum

**Affiliations:** 1 Department of Physiology, Faculty of Medicine, Khon Kaen University, Khon Kaen 40002, Thailand; 2 Department of General Practice, Khon Kaen Hospital, Khon Kaen 40000, Thailand; pudism@kkumail.com; 3 Department of Sport Science, Faculty of Education, Surindra Rajabhat University, Surindra 32000, Thailand; lertwanlop.s@srru.ac.th; 4 Department of Nursing, Praborommarajchanok Institute, Boromrajonani College of Nursing, Udonthani 42000, Thailand; s.wirakan@gmail.com; 5 Department of Physical Therapy, Faculty of Allied Health Sciences, Nakhon Ratchasima College, Nakhon Ratchasima 30000, Thailand; peerni@nmc.ac.th; 6 Cholangiocarcinoma Research Institute, Khon Kaen University, Khon Kaen 40002, Thailand; nattha.m@kkumail.com; 7 Faculty of Science and Technology, Uttaradit Rajabhat University, Uttaradit 53000, Thailand; worrawutt@uru.ac.th

**Keywords:** blood pressure, growth hormone, muscle cross-sectional area, venous occlusion, simulated altitude

## Abstract

Low-load resistance training combined with vascular occlusion or hypoxia can increase muscle cross-sectional area (CSA), but the effect of such training on hormonal response and cardiovascular response is less clear. Thirty female netball athletes took part in a 5-week training of knee muscles in which low-load resistance exercise (20% 1-RM) was combined with either an occlusion pressure (KT, n = 10), hypoxic air (HT, n = 10), or no additional stimulus (CT, n = 10). Growth hormones (GHs), cardiovascular parameters, and CSA were measured before and after the training program. Compared to CT, both HT and KT showed a substantial increase in GH release after the first training bout (pre). After 5 weeks of training (post), the release of GH was substantially reduced in all groups. Compared to CT, HT showed a substantial decrease in SP (11.7 ± 11.3%, mean ± 90% CL) over the training period. The reduction in systolic blood pressure (SP) after hypoxic training resulted in a substantial decrease in the rate-pressure product (RPP) by 15.6 ± 9.6%, compared to CT. CSA from HT and KT is likely related to the heightened release of GH found after training. The hypoxic training protocol has a greater cardiovascular benefit than similar resistance training with blood flow restriction.

## 1. Introduction

Strength training is a fundamental component of most sports conditioning programs, playing a key role in both injury prevention and rehabilitation. Research suggests that resistance levels of at least 60% of one repetition maximum (1RM) are necessary to stimulate strength improvements. However, for achieving optimal gains in muscular strength, training loads exceeding 80% of 1-RM are generally recommended [1]. While it is essential for building strength and performance, it also carries a risk of muscle and tendon injuries. Thus, low-load resistance training (20–50% 1-RM) combined with blood flow restriction or vascular occlusion can produce similar performance gains to traditional high-intensity resistance training [2] and may offer an alternative to resistance training for individuals who are unable to lift heavy weights [3]. However, the physiological mechanisms leading to muscle hypertrophy and strength gains that accompany low-load resistance training combined with limb blood flow restriction are not clear.

Postulated mechanisms thought to be involved in hypertrophy that accompanies low-load resistance training and blood flow restriction include hypoxia, cell swelling, and reactive oxygen species. Hypoxia causes increased lactate accumulation, which can increase the release of anabolic hormones and cytokines [4]. In addition, the increased reliance on anaerobic glycolysis under hypoxic conditions can lead to increased intramuscular lactate levels, which increase intracellular osmotic pressure [5]. Increased cellular hydration as a result of osmotic changes (cell swelling) has been associated with enhanced anabolic processes [6]. It is also possible that hypoxia-induced reactive oxygen species play a role in muscle hypertrophy [5], with nitric oxide being associated with satellite cell proliferation, which could theoretically lead to skeletal muscle growth [7].

Resistance training with blood flow restriction may increase discomfort [8,9,10] and may lead to low or retrograde blood flow, which has been shown to increase retrograde shear stress, a pro-atherogenic risk factor [11]. Previous research has shown that applying blood flow restriction (BFR) during low-load resistance training using single-exercise protocols of up to five sets performed to volitional failure does not appear to induce a muscle damage response [12].

Alternatively, systemic hypoxia (simulated altitude training) via a mask is a readily available and safe technique that reduces blood and muscle PO_2_ but allows unrestricted exercise without compromising blood flow. A recent study found that resistance training under hypoxic conditions effectively enhances muscle growth and strength, posing no additional risk compared to normoxic training [13]. In fact, previous research has found similar hypertrophy and strength gains in participants who performed low-load strength training under blood flow restriction or systemic hypoxia [14]

The mechanisms underlying muscle hypertrophy and strength gains from low-load resistance training remain insufficiently understood. Both blood flow restriction and systemic hypoxic training techniques are characterized by reduced oxygen supply [15]. However, hormonal responses to systemic hypoxia have only been examined in acute settings [16], and the immediate cardiovascular effects of these methods are not well established. Moreover, the safety of such training approaches for certain populations is still uncertain. Therefore, this study aimed to explore the distinct acute and chronic responses in growth hormone levels and cardiovascular function induced by low-load resistance training performed under either blood flow restriction or normobaric systemic hypoxia. We hypothesized that combining low-load resistance training with normobaric systemic hypoxia would result in a significantly greater cardiovascular response than training with blood flow restriction.

## 2. Materials and Methods

### 2.1. Participants

This study used a single-blind randomized controlled trial to determine the effect of 5 weeks of low-load resistance training with either vascular occlusion or systemic normobaric hypoxia on the acute and chronic hormonal and cardiovascular responses to such training.

**Figure 1:**
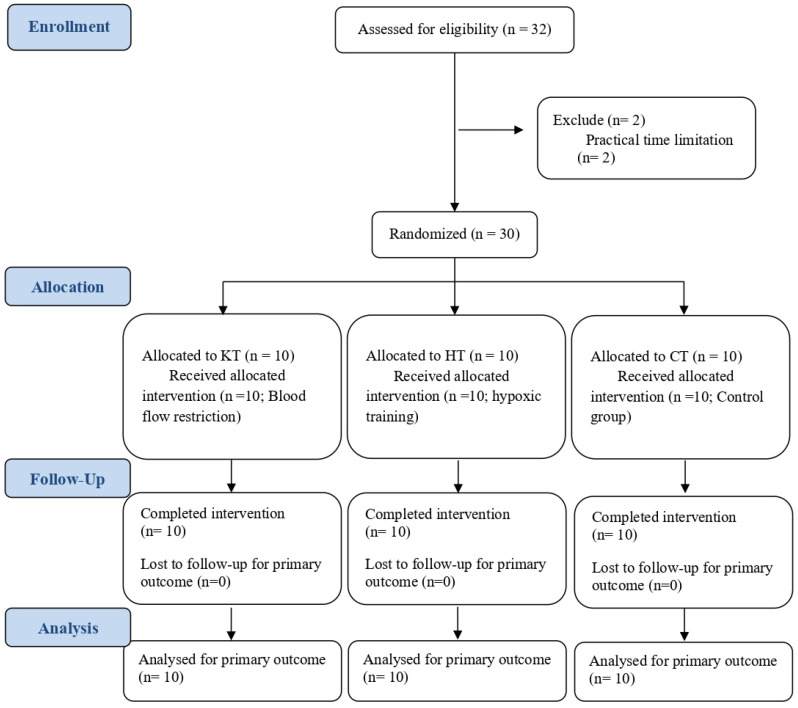
Consort diagram.

Thirty female university netball players (mean age: 20.2 ± 3.3 years; height: 168.4 ± 5.8 cm; body mass: 65.2 ± 6.5 kg) participated in this study (Figure 1). Each had approximately one year of experience competing at the university level. All participants provided written informed consent, and the study received approval from the University Human Ethics Committee. All participants were actively engaged in training, possessed comparable netball skills, underwent equal volumes of training, and were coached by the same physical conditioning specialist. Participants were instructed to fast and abstain from smoking and caffeine-containing beverages for 6 h prior to testing and avoid strenuous exercise for 24 h prior to testing.

### 2.2. Inclusion and Exclusion Criteria

All participating athletes satisfied the inclusion criteria as follows: they had no exposure to altitudes exceeding 1000 m within three months preceding the experiment; no history of severe acute mountain sickness; no health conditions contraindicating participation; and no use of medications such as anabolic steroids, creatine supplements, or sympathoadrenal agents during the study period. Exclusion criteria: Additionally, none of the athletes had engaged in resistance training within the preceding three months. Athletes were excluded if they presented with any medical conditions, such as hypertension, cardiovascular or pulmonary diseases, diabetes mellitus, musculoskeletal disorders affecting bones, joints, or muscles, or experiences of dizziness.

### 2.3. Protocol

**Figure 2:**
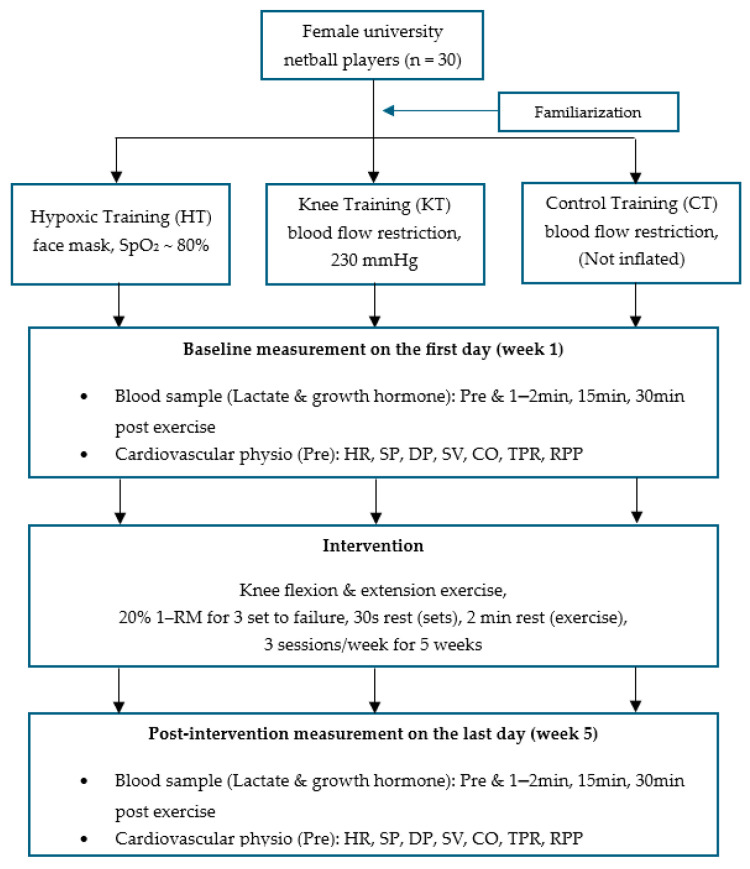
Experimental design.

Prior to the experimental period, all athletes underwent a comprehensive familiarization protocol with both the hypoxic and blood flow restriction (BFR) equipment, aligning with standard practice in these training modalities. During all training sessions, SpO_2_ and heart rate were continuously monitored in all athletes to mitigate any discomfort and prevent adverse effects associated with both training methods. Participants were randomly allocated into three groups using sealed envelopes, following a parallel group randomized controlled trial (RCT) design; blood flow restriction (KT, n = 10), hypoxic training (HT, n = 10), and no training control (CT, n = 10) (Figure 2). All participants carried out bilateral knee extensions and flexions within a 0° to 90° range; extensions were performed seated, and flexions were performed in a prone position using an isotonic leg extension and flexion machine (Fitness works Co., Ltd., Auckland, New Zealand) in an exercise laboratory. During both rest periods and exercise sets, individuals in the hypoxic training (HT) group were administered normobaric hypoxic gas via a face mask connected to a hypoxicator system (Airo HTMH; High Tech Mixing Head, Airo Limited, Co., Ltd., Auckland, New Zealand).

The hypoxicator employed a biofeedback control system to automatically regulate the fraction of inspired oxygen (FiO_2_), maintaining participants’ arterial oxygen saturation (SpO_2_) at approximately 80%, compared to a typical SpO_2_ of around 99%. The KT group trained using blood flow restriction (Kaatsu training) applied to both thighs via KAATSUMASTER^®^ mini cuffs (Sato Sports Plaza Inc., Tokyo, Japan), each approximately 5 cm wide. Cuff pressure was progressively increased by 10 mmHg daily from an initial 160 mmHg on Day 1 to a peak of 230 mmHg by Day 8, after which pressure levels remained consistent throughout the training. Heart rate and SpO_2_ were assessed at the end of each exercise set using a pulse oximeter (Sport-Stat, Nonin Medical, Minneapolis, Minnesota, USA). In contrast, the CT group conducted identical knee extension and flexion exercises while wearing non-inflated Kaatsu cuffs (<5 mmHg) and breathing ambient room air.

Participants engaged in a training program consisting of three sessions per week over a five-week period. Each session included three sets of knee extensions followed by three sets of knee flexions performed to failure, totaling six sets per session. Rest intervals were set at 30 s between sets and 2 min between exercises. On average, participants completed 28 ± 2, 24 ± 2 and 22 ± 2 repetitions per set for knee extensions and 36 ± 3, 31 ± 3, and 26 ± 3 repetitions per set for knee flexions (mean ± SD). Movements were executed at a consistent pace, with approximately 1 s allotted for both concentric and eccentric phases. Training resistance was standardized at 20% of 1 repetition maximum (1-RM), determined at least 2 days before the start of training and maintained throughout the study. To equalize training load across groups, HT and CT participants matched their repetition counts to those of the KT group. During training (~12–13 min per session), participants either inhaled the designated gas mixture or maintained blood flow occlusion via thigh cuffs, depending on group assignment.

### 2.4. Measurement

Cardiovascular variables: on week 1 (pre) and week 5 (post) of training, the heart rate (HR), systolic blood pressure (SP), diastolic blood pressure (DP), stroke volume (SV), cardiac output (CO), total peripheral resistance (TPR), and rate-pressure product (RPP) were measured by a Finometer Midi, Finapres Medical Systems, Enschede, Netherlands, at the end of each exercise set, which was performed by researcher.

Subjective scores on stress, fatigue, sleep, and training performance were recorded every day throughout the 5-week training period for monitoring the condition of the athletes.

Growth hormone and blood lactate levels: On the first and last day of training after an overnight fast, the participants rested for 30 min in the laboratory before a baseline blood collection. The venous blood samples were obtained from each participant’s forearm and were obtained pre-exercise (Pre) and 1–2 min, 15 min, and 30 min post-exercise for GH measurement. After collecting into a Vacutainer tube containing SST Gel and Clot Activator, the blood was allowed to clot at room temperature and was subsequently centrifuged at 1500× *g* for 15 min. The resulting serum was extracted and analyzed by a commercial laboratory technician (Medlab, Christchurch, New Zealand). In addition, blood lactate was determined at rest (Pre), 1–2 min, and 15 min post-exercise from a finger prick sample and analyzed using a portable analyzer (Lactate Pro, Arkray Inc., Kyoto, Japan).

Muscle cross-sectional area: Participants underwent magnetic resonance imaging (MRI) one week prior to and then 2–3 days following the five-week training regimen. Scans were performed using a Siemens 1.5 Tesla Avanto MRI system, Siemens Healthineers Co., Ltd., Belgique, Belgium. Bilateral coronal STIR (short-tau inversion recovery) images of the mid-thighs were captured using an expanded field of view. From axial slices taken at mid-thigh level, the cross-sectional areas of both knee flexor (biceps femoris) and extensor muscles (quadriceps) were manually delineated and analyzed using InteleRad PACS software, Intelerad Medical system. Each region of interest was outlined by hand, after which the software calculated the corresponding area. All measurements were conducted by a blinded examiner (medical doctor) in triplicate, with the average of the three readings used for analysis.

### 2.5. Statistical Analysis

We used a specifically designed spreadsheet available for controlled trials to calculate magnitude-based inferences about effect sizes (Bonetti et al.) [17] and then to make assumptions about the true (population) values of the effect. The uncertainty in the effect was expressed as 90% confidence limits (CLs). The probability that the true value of the effect was practically negative, trivial, or positive accounted for the observed difference and typical error of the measurement [18]. We generated the smallest worthwhile change value by multiplying the baseline between-subject standard deviation by Cohen’s value of the smallest worthwhile effect of 0.2 [19]. The chances that the true effects were substantial were estimated by the spreadsheet [18] when a value for the smallest worthwhile effect was entered. Effects that were simultaneously both >75% likely positive and <5% negative were considered substantial and beneficial. An effect was deemed unclear if its confidence interval overlapped the thresholds for substantiveness; that is, if the effect could be substantially positive and negative.

## 3. Results

**Figure 3:**
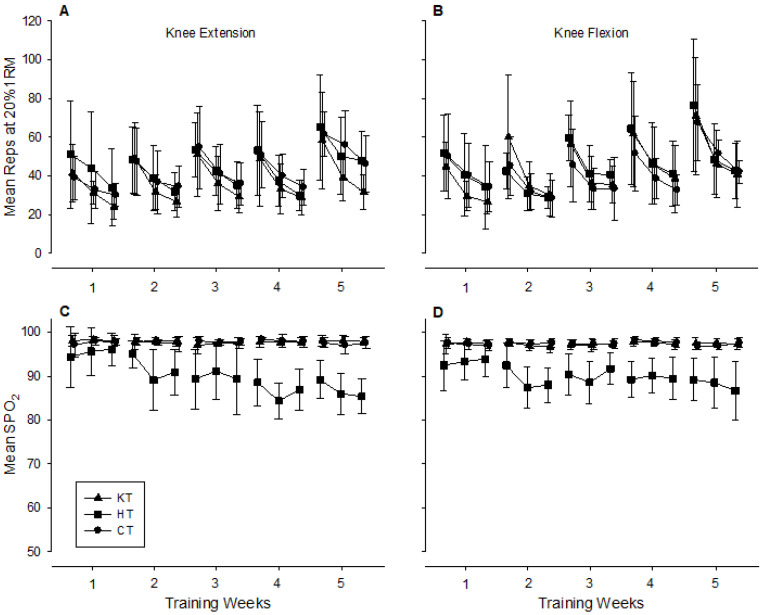
Average number of knee extension (**A**) and flexion (**B**) reps during the 3three sets on the last training day of each week and the accompanying arterial oxyhemoglobin saturation (SPO_2_) at the end of each of the three sets for the extension (**C**) and flexion (**D**) exercises.

The baseline characteristics, including age, body mass index, heart rate, and blood pressure (SP, DP) in all three groups, were presented; no significant differences existed, *p* > 0.05. The total amount of resistance work completed in all groups was similar and trended upwards over the 5 weeks of training (Figure 3A,B). The average number of reps per set increased by approximately 37% for knee extension and 34% for knee flexion over the course of the study (40 ± 22, 36 ± 15, and 39 ± 16 reps per set on day 1 and 54 ± 16, 48 ± 23, and 55 ± 24 reps per set on day 15 for the CT, KT, and HT groups, respectively). There was little difference in the SPO_2_ at the end of each set over the course of the study in the CT or KT groups, but as training progressed, the SPO_2_ decreased to a greater degree in the HT group (Figure 3C,D).

**Figure 4:**
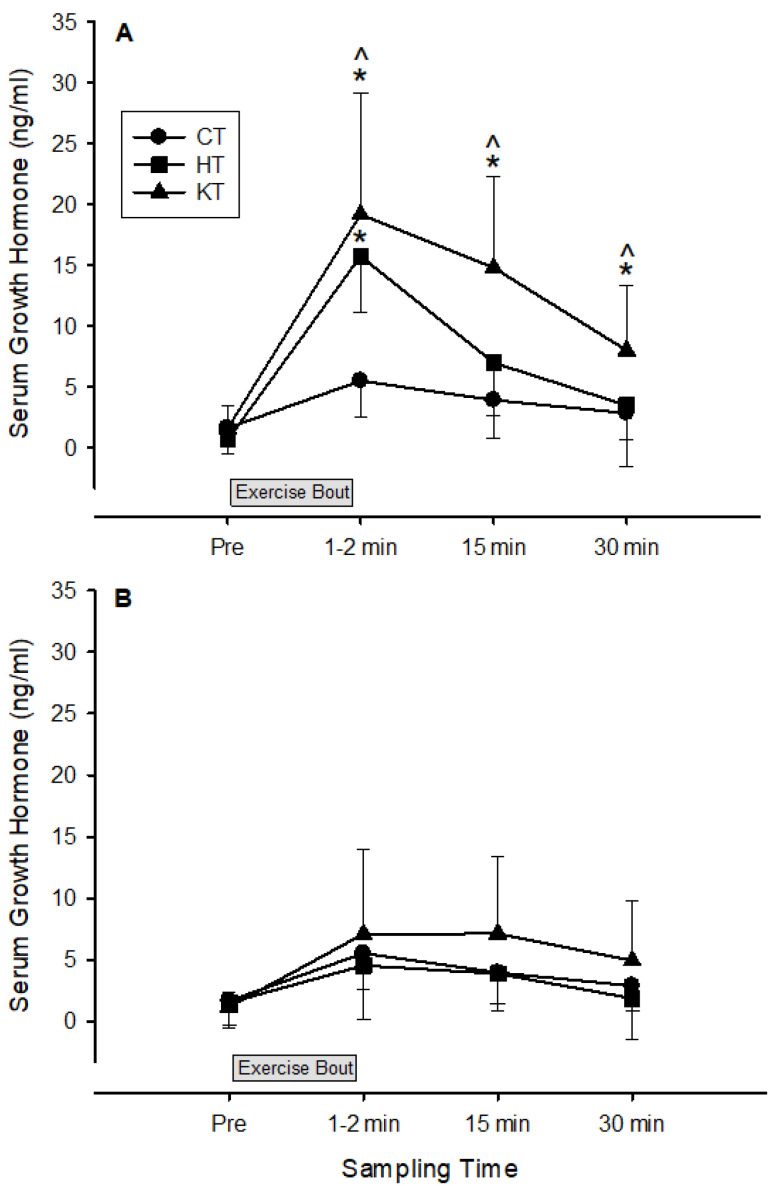
Changes in serum growth hormone as a result of acute (first training day, (**A**)) and chronic (last training day, (**B**)) low-load resistance training. * Substantially different from the CT group. ˄ Substantially different from the HT group.

Compared to the control group, both the hypoxic and venous occlusion groups showed a large increase in exercise-induced serum growth hormone release 1–2 min after the first training bout (6.1 ± 3.7 and 6.4 ± 1.9 mean Cohen’s d ± 90% confidence interval for the hypoxic and venous occlusion group, respectively). Growth hormone release continued to be substantially elevated in the venous occlusion group compared to the control and hypoxic groups up to 30 min post the first training bout (Figure 4A). After 5 weeks of low-load resistance training, the release of serum growth hormone post-exercise was substantially reduced and similar in all groups (Figure 4B). The area under the curve for exercise-induced growth hormone response decreased substantially in both experimental groups as a result of the 5 weeks of low-load resistance training (baseline: 23.6 ± 6.3 and 39.5 ± 21.2 ng/mL; post-training: 10.1 ± 7.1 and 15.3 ± 14.2 ng/mL for the HT and KT groups, respectively, mean ± SD). 

Compared to the control group, the change in CSA in the KT group after the 5-week low-load resistance training was significantly higher in both extensor and flexor muscle groups, while the HT group showed a significant increase in only the extensor muscle groups (Table 1). 

Relative to the control group, the percentage change in SP in the HT group after the 5-week low-load resistance training was significantly lower (11.7 ± 11.3%, mean ± 90% CL). Although there was a significant increase in DP (9.8 ± 10.8%), MAP (5.7 ± 6.5), and TPR (68.8 ± 40.4) in the KT group at the end of the last set of the training protocol, SV (17.2 ± 19.5%) and CO (18.0 ± 21.9%) decreased. The rate-pressure product (RPP) showed a significant decrease in the HT groups (15.6 ± 9.6%, mean ± 90% CL) but no significant decrease in the KT groups (3.9 ± 14.3%) compared to CT (Table 2). 

**Figure 5:**
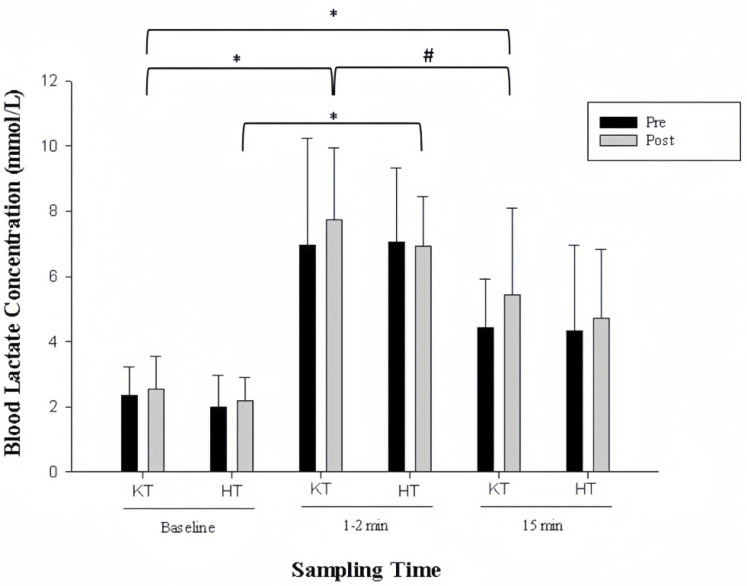
Changes in blood lactate levels at the first training day (Pre: dark bar) and last training day (Post: light bar). KT: venous occlusion group; HT: hypoxic group. * Significantly different from baseline. ^#^ Significantly different between 1–2 min and 15 min post-acute bout of exercise.

Blood lactate concentration increased approximately 3-fold, 1–2 min after the first training bout in all groups, with little difference between groups (Figure 5). After 5 weeks of training, the concentration of blood lactate post-exercise in the hypoxic and venous occlusion groups was similar to pre-training levels, with differences between the groups unclear at all sampling times. The blood lactate response in the two intervention groups showed little change as a result of the 5 weeks of low-load resistance training (baseline: 11.2 ± 3.2 and 10.4 ± 4.03 mmol/L; post-training: 11.0 ± 1.5 and 11.3 ± 2.6 mmol/L for the HT and KT groups, respectively, mean ± SD).

## 4. Discussion

In the current study, we have demonstrated that low-load resistance exercise in combination with systemic hypoxia or blood flow restriction caused larger elevations in blood lactate and serum growth hormone concentrations immediately after exercise compared to a normoxic control group. These results suggest that low-load resistance training under blood flow restriction or systemic hypoxic conditions evokes a greater metabolic stress and hormonal response compared to similar resistance training under normoxic conditions. Five weeks of such training resulted in enhanced muscle hypertrophy but reduced exercise-induced growth hormone response.

Compared to CT group conditions, 5 weeks of low-load high repetition exercise training with either blood flow restriction or systemic hypoxia resulted in considerable muscle hypertrophy (7.6 and 5.3% in the KT and HT groups, respectively). Similar changes in CSA have been reported in previous strength training studies using either systemic hypoxia (7.3%) [20] or vascular occlusion (6.3%) [21] and are compatible with traditional high-intensity (~80%1RM) strength training (6.1%) [21] (12.6%) [22]. In contrast, Friedmann et al. (2003) reported no significant increase in CSA in a group of untrained males after undergoing 4 weeks of low-load high repetition strength training in systemic hypoxia (FIO_2_ = 0.12) [23]. However, unlike the current study, Friedmann and associates did not have participants breathe hypoxic air between sets during recovery, thereby almost halving the time under hypoxia. Importantly, since the participants in the current study exercised to exhaustion (or matched the number of reps completed by the KT group working to exhaustion), the total amount of work completed by the participants of this study was considerably more than that of the Friedmann study (6 sets of 25 reps and 6 sets of ~37–47 reps in the Friedmann and current study, respectively). The larger exercise-induced growth hormone response observed in the HT and KT groups after the first training session may have played a key role in mediating the observed muscle hypertrophy [24]. The higher workload probably resulted in increased stress, resulting in greater adaptation and subsequent muscle hypertrophy.

Serum growth hormone concentration followed a similar response to blood lactate concentration and reached an apex 1–2 min post-exercise, where it was three- to four-fold higher in the HT and KT groups compared to the CT group at the beginning of the 5-week training bout (Figure 3A). It has been suggested that the acute growth hormone response to resistance training is influenced by the metabolic stress of the workout, such that exercise bouts that elicit high levels of blood lactate also tend to produce high levels of growth hormone [25]. It is thought that the increased H^+^ concentration produced by lactic acidosis is a major factor influencing growth hormone release [26]. Previous research has confirmed high correlations between blood lactate and growth hormone concentrations after high-intensity resistance training [27]. In contrast, reducing post-exercise blood H^+^ accumulation via sodium bicarbonate ingestion subsequently reduced growth hormone release [28]. Furthermore, the reduced-exercise-induced GH response after 5 weeks of training in the HT and KT groups may reflect a potential down-regulation of the somatotropic axis in response to the repeated high metabolic stress imposed by these training modalities. This down-regulation may be a protective mechanism to avoid excessive catabolism and allow for optimal recovery and adaptation [24,29,30].

Low-load resistance training under hypoxia led to a decrease in systolic blood pressure during exercise, whereas the group with blood flow restriction had no effect. The reduction in SBP in the hypoxia group was associated with a lower rate-pressure product (RPP), indicating reduced myocardial oxygen demand [31]. Previous studies reported reductions in SBP and DBP after training at moderate altitude [32] and after a 3-week exposure to 1700 m altitude [33] in both normotensive and hypertensive groups. This physiological adaptation could be due to the up-regulation of vascular reactivity, particularly morphological changes in the endothelium and possibly a reduction in sympathetic activation [34]. Lyamina et al. (2011) found increased nitric oxide synthesis and a reduction in blood pressure in hypertensive individuals to levels similar to normotensive individuals [35]. Hypoxia-induced NO release reduces total peripheral resistance, lowering SBP [35], whereas BFR maintains high sympathetic nervous system (SNS) activity, preventing SBP reduction [36]. Our data suggests that cardiac workload was lower during systemic hypoxic low-load resistance training than during localized hypoxia due to blood occlusion in netball athletes [35]. Indeed, hypoxic training may have improved cardiovascular health by lowering systolic blood pressure during exercise, whereas blood flow restriction training has detrimental effects on the cardiovascular system during exercise. However, the mechanism behind the SBP changes needs further investigation.

The current study supports the strong association between blood lactate accumulation and growth hormone response after acute resistance exercise; however, this is not the case after chronic training. The current study shows that after 5 weeks of low-load resistance training with either systemic hypoxia or venous occlusion, the post-exercise blood lactate response remains relatively high and similar to pre-training levels, but the growth hormone response is substantially reduced. The growth hormone response to acute resistance exercise has been reported to be lower [37], unchanged [38], or higher [39] post-training. Differences in growth hormone response between training studies are likely to be associated with various factors, including the number of growth hormone samples collected (single vs. multiple), when the samples were collected (to account for the phasic response after exercise), and how the samples were analyzed (e.g., area under the curve or individual sample points) [40]. In addition, the relative intensity of training is invariably associated with growth hormone release [41,42], which may explain why the participants in the current study showed a decreased exercise-induced response after 5 weeks of training. Even though the absolute workload increased in all groups (Figure 3A,B), the subjective stress of the training decreased over time, probably due to improvements in muscular fitness. The systemic hypoxic intervention maintained and, indeed, increased the hypoxic environment in the muscles (see SPO_2_ in Figure 3C,D) as the training continued, presumably due to increased time to complete the reps of each set. Although we were unable to measure SPO_2_ past the occlusion site in the legs, we assume a similar trend occurred in the venous occlusion group as the participants became stronger and completed more reps per set towards the end of the training period. The continued hypoxic environment in these two groups resulted in a relatively high blood lactate response on the last training day in the face of decreased overall training stress (as per subjective ratings). We hypothesize that growth hormone release is associated more with the physical stress of the exercise than with hypoxia-induced blood lactate and H^+^ concentration. Takarada et al. (2000) found a significant increase in plasma growth hormone concentration after training. This suggests that the enhanced growth hormone response is linked to acute physical stress imposed by the combination of exercise and vascular occlusion [4].

Peak blood lactate concentrations in the HT and KT groups were approximately three-fold higher than those in the CT group. Exercise under venous occlusion conditions [17] or systemic hypoxia [16] has been shown to significantly lower blood oxygenation levels at the muscle, which would result in an increase in the anaerobic metabolism of ATP, leading to higher blood lactate accumulation. Interestingly, as a result of acute low-load resistance training, blood lactate concentration in the HT group remained constant after the training (approximately 8 mmoL/L) bout compared with KT and CT, but after 5 weeks of training, blood lactate concentration in the HT group reduced to approximately 5 mmoL/L 15 min post-training. This may indicate that buffering capacity plays a role after 5 weeks of training in the HT group but not in the KT group, which was not changed after training. If this is the case, anaerobic capacity may increase as a result of this exercise under hypoxic conditions rather than venous occlusion. Our previous study reported that muscle strength-endurance (number of repetitions: Reps 20 of 1RM) in an HT group increased more than in a KT group (74.1 ± 82.1 vs. 61.5 ± 27.1) after training. This correlated with the reduction in blood lactate level in HT after training in the current study (lactate threshold may increase). This previous study showed a rough picture of hypoxia with resistance exercise, which trended to improve muscle strength-endurance, while venous occlusion with resistance training trended to improve hypertrophy and peak force (maximum voluntary contraction in 3 s: MVC3) [14].

## 5. Conclusions

The results suggest that low-load high repetition resistance training with venous occlusion or hypoxia results in muscle hypertrophy levels similar to traditional high-intensity resistance training. Our result suggests (at least after training) that the release of growth hormone is less related to the H^+^ concentration and more related to the physical stress of the exercise bout. Resistance training with low loads in combination with systemic hypoxia causes less harm in terms of blood pressure changes and cardiac workload during exercise compared to local hypoxia produced by blood occlusion. 

Limitation: This research does not address the long-term outcomes of low-load resistance training conducted under hypoxic conditions or with blood flow restriction. To gain clearer insights, future studies should evaluate post-training effects over a minimum duration of one week.

Practical implication: Hypoxic training appears to enhance cardiovascular health by reducing systolic blood pressure during exercise, whereas blood flow restriction (BFR) training may have less impact on cardiovascular function under similar conditions. This suggests that hypoxic training might improve buffering capacity, potentially leading to greater anaerobic capability compared to BFR training. However, BFR training demonstrates superior effectiveness for increasing muscle mass. Therefore, athletes and coaches can select the appropriate training method based on their primary performance goals. Moreover, individuals who aren’t athletes can use hypoxic training to enhance cardiovascular health and anaerobic performance, often pairing it with various low-intensity workouts.

## Figures and Tables

**Table 1:** Differences in the changes between groups and chances that the true differences represent a substantial improvement in muscle cross-sectional area (cm^2^) in KT and HT compared to CT.

Muscles	Effect of KT (KT vs. CT) (cm^2^, ±90% CL)	Practical Outcome	Effect of HT (HT vs. CT) (cm^2^, ±90% CL)	Practical Outcome
Extensors				
Rectus femoris	2.1 ± 1.6	Very likely	0.7 ± 1.1	Unclear
Vastus lateralis	1.7 ± 1.6	Likely	0.4 ± 0.9	Unclear
Vastus medialis	1.7 ± 1.5	Likely	0.4 ± 1.2	Unclear
Flexors				
Biceps femoris	0.7 ± 0.8	Likely	1.3 ± 0.9	Very likely
Semitendinosus	1.1 ± 0.8	Very likely	1.1 ± 0.8	Very likely
Semimembranosus	0.7 ± 0.7	Likely	1.4 ± 0.8	Very likely

Data are mean ±90% confidence limits in cm^2^ units; add and subtract this number from the mean effect to obtain the 90% confidence limits for the true difference. CT: control training; KT: Kaatsu training; HT: hypoxic training.

**Table 2:** Differences in the changes between groups and chances that the true differences represent a substantial improvement in cardiovascular parameters in KT and HT compared to CT.

Parameters	Effect of KT (KT vs. CT) (±90% CL)	Practical Outcome	Effect of HT (HT vs. CT) (±90% CL)	Practical Outcome
Systolic (mmHg)	−1.8 ± 8.9	Unclear	−11.7 ± 11.3	Almost certainly
Diastolic (mmHg)	9.8 ± 10.8	Likely	1.6 ± 12.5	Unclear
Mean (mmHg)	5.7 ± 6.5	Likely	2.4 ± 11.7	Unclear
Heart Rate (bpm)	−2.4 ± 15.1	Unclear	1.3 ± 12.1	Unclear
Stroke Volume (mL)	−17.2 ± 19.5	Likely	−1.2 ± 19.7	Unclear
LVET (ms)	−3.8 ± 6.9	Unclear	4.6 ± 5.9	Very likely
Pulse Interval (ms)	0.5 ± 17.1	Unclear	−2.8 ± 13.8	Unclear
Maximum Slope (mmHg/s)	1.2 ± 18.3	Unclear	8.3 ± 27.4	Unclear
Cardiac Output (L/min)	−18.0 ± 21.9	Likely	6.4 ± 23.9	Unclear
TPR (dyn·s/cm^5^)	68.8 ± 40.4	Very likely	−1.3 ± 37.0	Unclear

Data are mean ± 90% confidence limits; add and subtract this number from the mean effect to obtain the 90% confidence limits for the true difference. CT: control training; KT: Kaatsu training; HT: hypoxic training.

## Data Availability

The data presented in this study are available upon request from the corresponding author.

## Abbreviations

The following abbreviations are used in this manuscript:

CSA	Cross-Sectional Area
1-RM	1 Repetition Maximum
MVC	Maximal Voluntary Contraction
SBP	Systolic Blood Pressure
DBP	Diastolic Blood Pressure
MAP	Mean Arterial Pressure
SV	Stroke Volume
LVET	Left Ventricular Ejection Time
NO	Nitric Oxide
